# Interventions to improve neonatal and infant intubation success: a meta-analysis

**DOI:** 10.1007/s00431-026-07119-7

**Published:** 2026-05-29

**Authors:** Wenla Tjurin, Panu Kiviranta, Heli Salmi, Ilari Kuitunen

**Affiliations:** 1 Kuopio Pediatric Research Unit, University of Eastern Finland, Kuopio, Finland; 2 Department of Pediatrics and Neonatology, Kuopio University Hospital, Puijonlaaksontie 2, 70211 Kuopio, Finland; 3 Finnish Medical Society Duodecim, Helsinki, Finland; 4 Department of Anaesthesiology, Intensive Care and Pain Medicine, New Childrens Hospital, University of Helsinki and Helsinki University Hospital, Helsinki, Finland

**Keywords:** Endotracheal intubation, Interventions, Airway, Infant

## Abstract

The aim of this study is to synthesize and evaluate the effectiveness of interventions aimed at improving endotracheal intubation success in neonates and infants. PubMed, Scopus, and Web of Science were searched from inception through July 2025. Randomized studies involving neonatal or infant endotracheal intubation were included regardless of intervention or comparator. Two reviewers independently extracted data and assessed risk of bias. Data were pooled using a random-effects inverse-variance model. Results are reported as risk ratios (RRs) with 95% confidence intervals (CIs). Certainty of evidence was assessed using the GRADE approach. Thirty studies published between 2002 and 2025 were included from 287 screened records. Most intubations were elective and performed in neonatal intensive care units or operating rooms. Video laryngoscopy improved first-attempt success compared with direct laryngoscopy (RR 1.13; 95% CI 1.06–1.20; high-certainty evidence). Pharmacologic interventions, particularly neuromuscular blocking agents and sedatives, generally increased success. Noninvasive respiratory support during intubation improved success (RR 1.18; 95% CI 1.02–1.38; moderate-certainty evidence). Time to successful intubation was similar across most interventions, except for neuromuscular blockade, which shortened intubation duration. Desaturation-related outcomes did not differ. *Conclusion*: Video laryngoscopy improves first-attempt intubation success with high-certainty evidence, and noninvasive respiratory support most likely improves success. These findings support their use during neonatal and infant intubation.

**Table:** 

**What is Known:** • *Intubation in neonates and infants carries a high complication risk, making fi rst-attempt successcritical.* • *Video laryngoscopy and pharmacological premedication each improve intubation conditions but have previouslybeen evaluated in isolation. *
**What is New:** • *Pooled randomized evidence confi rms with high certainty that video laryngoscopy improves fi rst-attempt success over direct laryngoscopy without prolonging intubation time. * • *Non-invasive respiratory support during intubation most likely improves fi rst-attempt success and physiologicalsafety.*

**Supplementary Information:**

The online version contains supplementary material available at 10.1007/s00431-026-07119-7.

## Introduction

Complications during intubation are common, particularly in neonates and infants. In neonates, intubation is challenging due to factors such as small size, low weight, and unique anatomy [1]. In addition to these anatomical difficulties, neonates are physiologically vulnerable, with limited oxygen reserves. Consequently, desaturation occurs rapidly, and securing the airway must be accomplished quickly [2]. Reported complications include hypoxemia, bradycardia, hypertension, intracranial hemorrhage, and even death [1].

Over recent decades, the frequency of neonatal intubations has declined substantially driven by improved antenatal care, advances in non-invasive ventilation, and the development of neonatal resuscitation programs [3]. For many decades, direct laryngoscopy was the standard technique for intubation. However, video laryngoscopy has demonstrated higher first attempt success rates. Achieving success on the first attempt is critical, as the risk of complications increases with each additional attempt [4]. Video laryngoscopy provides improved visualization of anatomical structures and appears to improve both success rates and safety in neonatal and infant intubation [5–7].

Neonates are often intubated in critical situations with inadequate anesthesia and neuromuscular blockade. Several trials have shown that premedication improves intubation conditions and reduces both procedure time and the number of attempts [8]. Ideally, premedication should eliminate pain and discomfort, facilitate intubation, minimize the risk of traumatic injury, and avoid adverse effects. No current analgesics fully meet these criteria, and numerous studies have explored different options for pharmacological agents [9].

International guidelines recommend routine use of video laryngoscopy and pharmacological agents [10]. Several previous systematic reviews have examined specific interventions aimed at improving neonatal intubation success, most commonly focusing on video laryngoscopy or pharmacological premedication [6, 11]. However, these reviews have typically evaluated individual techniques rather than providing a comprehensive overview of all available randomized evidence. As a result, clinicians lack a unified synthesis comparing the effectiveness of different strategies used to improve intubation success in neonates and infants. In addition, newer approaches such as the use of non-invasive respiratory support during intubation have been evaluated in recent randomized trials but have not been comprehensively synthesized alongside other airway management strategies [12].

To provide an updated synthesis, we conducted a systematic review and meta-analysis of all interventions evaluated in these trials comparing the outcomes including first attempt success rate, time to successful intubation, and desaturation rate.

## Methods

### Search and study selection process

We conducted systematic review and meta-analysis, searching PubMed, Scopus, and Web of Science databases in July 2025. The review was reported in accordance with the Preferred Reporting Items in Systematic Reviews and Meta-Analyses (PRISMA) guidelines, and the checklist is provided in the supplementary materials [13]. The complete search strategy is also available in the supplementary materials. Search results were imported to Covidence software (Veritas Healthcare Inc, Victoria, Australia) for screening. Two authors (WT and IK) independently screened the abstracts and full-reports; disagreements were resolved by consensus. We used Cochrane’s automated randomized controlled trial (RCT) classifier tool during screening, which excluded studies identified as non-randomized [14].

### Eligibility criteria

We included studies on neonate and infant intubation. Neonates were defined as children aged 0–28 days, or preterm neonates prior to reaching a corrected gestational age 42 + 0 weeks. Infants were classified as children aged 0–365 days. Eligible intervention targeted either the provider or patient, with no restriction on intervention or comparator. Outcomes of interest were intubation success, time to intubation, or desaturation. Only parallel-grouped individually randomized controlled trials were included; crossover and cluster-randomized trials were excluded. Observational studies, animal studies, and manikin studies were excluded. Studies that included infants or neonates but did not report stratified results for these age groups were also excluded.

### Data extraction

Two authors initially extracted data from 20% of the included studies; as no discrepancies were noted, a single author (WT) completed the remaining extractions, which were verified by a second author (IK). Data were reported in a pre-designed Excel spreadsheet. Extracted information included the following: authors, study years, country, inclusion/exclusion criteria, intervention and comparator details, patient and provider characteristics, first- attempt success rate, time to successful intubation, desaturation outcomes, funding, and conflict of interest disclosures.

### Outcomes

The primary outcome was the first attempt intubation success. Secondary outcomes included time to successful intubation and desaturation (event rate or duration or lowest measured saturation during intubation).

### Interventions

We classified the interventions included for analysis as follows: video laryngoscopy vs direct laryngoscopy, video laryngoscope type comparisons, digital intubation, premedication strategies, neuromuscular blockade, and noninvasive respiratory support strategies during the intubation.

### Risk of bias and certainty of evidence

Risk of bias was assessed using the Cochrane Risk of Bias 2.0 tool [15]. For the assessment, we classified the three outcomes (successful intubation, time to intubation, and desaturation) as objective outcomes. Thus, we have reported a single risk of bias assessment per each study and have not automatically rated the risk of bias high due to lack of blinding [16].

Certainty of evidence was assessed using the core GRADE framework [17]. GRADE assessment was performed for each comparison with statistical pooling of the results and only for the main outcome of first attempt success rate. The certainty of evidence was rated from very low to high. Comparisons based on a single study were rated as very low due to imprecision. Imprecision was not automatically downgraded for confidence interval crossing one. Instead, a contextualized approach was applied, considering a relative effect of 10% as clinically meaningful [18]. Inconsistency was assessed using the *I*^2^ statistic and visual inspection of forest plot asymmetry [19].

### Statistics

Meta-analyses were performed using RevMan web, and summary plots were generated using Python (matplotlib). Analysis followed Cochrane handbook of systematic review guidelines. For the primary outcome, studies with similar interventions and controls were pooled together, when at least two trials reported the same outcome. We used DerSimonian and Laird inverse variance random-effect meta-analysis with Wald estimation to calculate risk ratios (RR) with 95% confidence intervals (CI). For the secondary outcome of time to successful intubation, we used DerSimonian and Laird inverse variance random-effects model to calculated mean difference (MD) with CI. We calculated the means and SD from studies reporting median and interquartile range according to the guidance of the Cochrane handbook. Random-effects models were applied throughout due to anticipated heterogeneity in the study populations and study settings. The inconsistency index statistic *I*^2^ for heterogeneity was also conducted and presented alongside the forest plots. Publication bias was assessed visually using funnel plots. Desaturation we have followed the synthesis without meta-analysis (SWiM) guidance and have reported these finding as narratively [20]. Sensitivity analysis with only low risk of bias studies included were performed to address the impact of study limitations on the effect estimates. Meta-regression was considered but not performed, as only the video-versus-direct-laryngoscopy comparison contained a sufficient number of studies (≥ 10) for reliable adjustment, and the relevant effect modifiers (clinical setting, indication, and intubator experience) for that comparison have been examined in a previous subgroup meta-analysis by our group [6].

### Protocol

The review protocol was registered to Open Science Framework and is freely available at: https://osf.io/hnd86/overview

## Results

### Search results and study characteristics

**Fig. 1:**
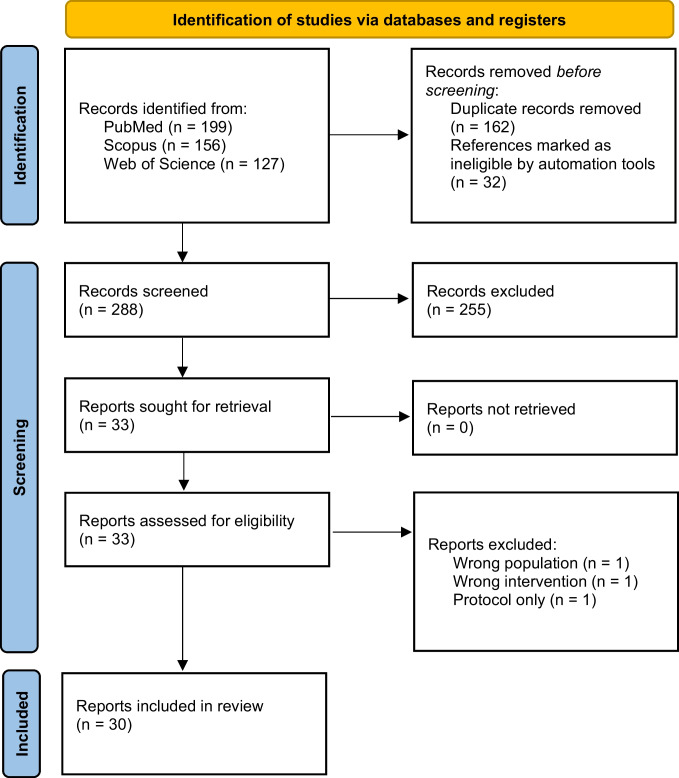
PRISMA flowchart of the study selection process

A total of 288 records were screened, 33 studies were assessed in full, and 30 studies [4, 5, 12, 21–46] were included in the review (Fig. 1). The included studies were published between 2002 and 2025 (Table 1). Eight studies were conducted in Asia, six studies in Europe, six in North America, three in Australia, two in Africa, and one in South America; four were multinational (Table S1). Intubation was performed in NICUs in 13 studies, operating room in 11 studies, delivery rooms or NICUs in five studies, and one study included participants from both NICU and operating room settings (Table 1). Nineteen studies focused on elective intubations, whereas eleven included acute intubations (Table 1).

**Table 1:** Characteristics of the included studies

Study	Study setting	Age criteria	Indication	Intubation position	Intubator	Intervention	Control
Video laryngoscopy vs direct laryngoscopy							
Abdalla 2025	Operating room	0–12 months	Elective	Back lying	Anesthesiologist	C-MAC video laryngoscopy	Direct laryngoscopy
Fiadjoe 2012	Operating room	0–12 months	Elective	Back lying	Anesthesiologist	Glide-Scope Cobalt video laryngoscope	Direct laryngoscopy with a Miller blade
Garcia-Marcinkiewicz 2020	Operating room	0–12 months	Elective	Back lying	Anesthesiologist	Video laryngoscopy with a standard blade	Direct laryngoscopy
Geraghty 2024	Delivery room and NICU	Neonates of any gestational age	Acute	Back lying	doctors training in pediatrics and neonatology,	Video laryngoscopy	Direct laryngoscopy
Goel 2022	Operating room	0–28 days	Elective	Back lying	Anesthesiologist	C-MAC video laryngoscopy	Miller laryngoscope
Jain 2018	Operating room	0–12 months	Elective	Left lateral position	Anesthesiologist	C-MAC miller video laryngoscope	Direct Miller laryngoscope
Moussa 2016	NICU	Newborn infants	Acute	Back lying	Junior peadiatric residents	Video laryngoscope	Classic laryngoscope
Salama 2019	Operating room	Age ≤ 28 days	Elective	Lateral position	Anesthesiologist	Glide-Scope video laryngoscope	Miller direct laryngoscope
Riva 2023	OR or NICU	< 52 weeks	Elective	Back lying	Anesthesiologist	Video laryngoscope	Direct laryngoscope
Tao 2019	Operating room	Age ≤ 28 days	Elective	Back lying	Anesthesiologist	GlideScope video laryngoscope	Direct laryngoscope
Tippmann 2023	Delivery room or NICU	Neonates < 44 weeks	Acute	Back lying	Mixed providers	Video laryngoscope	Direct laryngoscope
Venkatesh 2025	Operating room	Neonates and infants 6 months of age	Elective	Back lying	Anesthesiologist	Besdata video laryngoscope	Miller direct laryngoscope
Volz 2018	NICU	Neonates and infants	Elective	Back lying	Second year residents	video during direct laryngoscope	traditional direct laryngoscope
Video laryngoscopy model comparisons							
Gupta 2018	Operating room	Neonates and infants	Elective	Back lying	Anesthesiologist	C-MAC video laryngoscope	the Truview Picture Capture Device
Gupta 2021	Operating room	Neonates and infants	Elective	Back lying	Anesthesiologist	C-MAC Miller video laryngoscope	McGrath MAC videolaryngoscope
Medication strategies							
Avino 2014	NICU	Gestational age ≥ 28 weeks	Elective	Back lying	Neonatalogist	Remifentanil	Morphine-midazolan
Choong 2010	NICU	Term and preterm neonates	Elective	Back lying	Mixed providers	Remifentanil	Fentanyl
Feltman 2011	NICU	Term and preterm neonates	Elective	Back lying	Mixed providers	Rocuronium, atropine, fentanyl	Atropine, fentanyl
Ghanta 2007	NICU	Infants	Elective	Back lying	Mixed providers	Propofol	Morphine, atropine, and suxamethonium
Milési 2018	Delivery room and NICU	Preterm neonates	Acute	Back lying	Pediatrician	Midazolam	Ketamine
Oei 2002	NICU	25–40 weeks old	Acute	Back lying	Junior medical staff	Premedication	Awake
Roberts 2006	NICU	Infants	Elective	Back lying	Mixed providers	Mivacurium, Atropine, fentanyl	Atropine, fentanyl
Noninvasive respiratory support during intubation							
Foran 2023	NICU	Term and preterm infants	Acute	Back lying	Mixed providers	NHF (6 L/min and FiO2 of 1.0)	NHF with no flow
Hodgson 2022	Delivery room and NICU	Newborns	Acute	Back lying	Mixed providers	Nasal high flow therapy	Standard care
Ilhan 2025	NICU	Infants with a gestational age of 22–41 weeks	Elective	Back lying	Residents	Nasal Intermittent Positive Pressure Ventilation	Standard care
Other							
Kamlin 2013	Delivery room and NICU	Newborn infants	Acute	Back lying	Pediatric residents or fellows	Stylet	No stylet
Moura 2006	NICU	Newborn infants	Acute	Back lying	Researcher	Digital intubation	Direct laryngoscope intubation
O'Shea 2015	NICU	Newborn infants	Acute	Back lying	Doctors < 6 months experience in the NICU	The video laryngoscope screen visible to the instructor	The video laryngoscope screen covered
Park 2023	Operating room	0–12 months	Elective	Back lying	Anesthesiology residents	C-curved stylet	The hockey stick curved stylet
Roberts 2006	NICU	Infants	Elective	Back lying	Mixed providers	Mivacurium, Atropine, fentanyl	Atropine, fentanyl
Solanki 2024	NICU	Neonates in the NICU	Acute	Back lying	Pediatric surgery residents	Endotracheal tube with stylet	Endotracheal tube without stylet

Twenty-eight studies used the supine position for intubation and two used a lateral position. The experience level of the intubating physician varied considerably across studies. Fourteen studies compared video laryngoscopy with direct laryngoscopy, two studies compared different video laryngoscopes, and one compared digital intubation to direct laryngoscopes (Table 1). Seven studies compared different induction of general anesthesia. Three studies focused on respiratory management approaches, including high flow nasal cannula (HFNC) and non-invasive positive pressure ventilation (NIPPV). Three studies compared different stylet configurations. The patient characteristics are presented in Table S1.

### Risk of bias

**Fig. 2:**
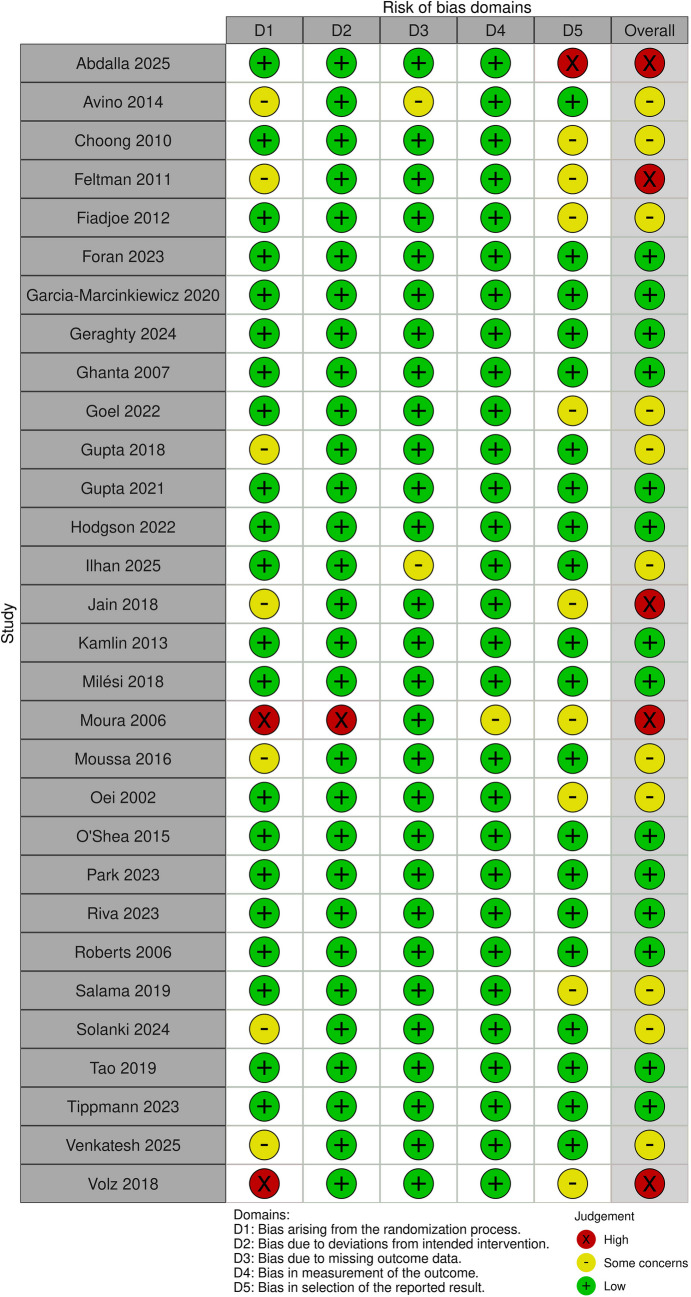
Risk of bias in the included studies assessed according to the Cochrane risk of bias 2.0 tool and all outcomes considered as objective in the assessment

The overall risk of bias was rated as low in fourteen, had some concerns in eleven, and was high in five studies (Fig. 2). Most issues arose from the randomization process and selective reporting.

### First attempt success rate

**Fig. 3:**
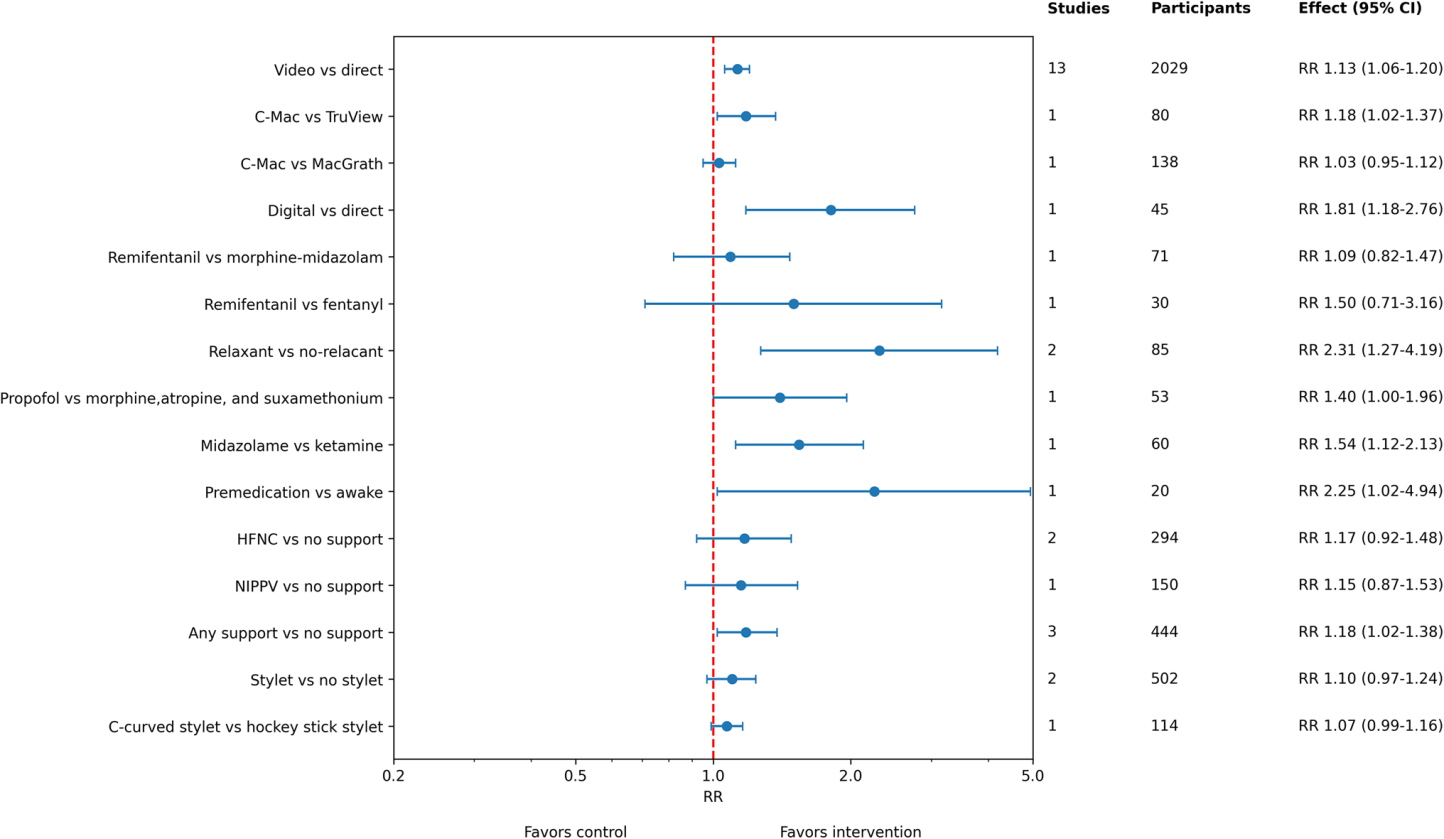
Summary forest plot of the comparisons for the outcome of first attempt intubation success rate in neonates and infants

Figure 3 summarizes pooled effect estimates. First-attempt success was analyzed in 29 studies. Among 14 studies comparing video-laryngoscopy with direct laryngoscopy, 13 (2029 patients) reported first attempt success rates, which were higher with video laryngoscopy than in the direct laryngoscope group (RR 1.13, CI 1.06–1.20; Figure S1). Certainty of evidence was rated as high (Table 2). Sensitivity analysis restricted to low-risk-of-bias studies yielded a similar effect estimate (RR 1.17, CI 1.04–1.31; Figure S2). Funnel plot inspection revealed no signs of publication bias (Figure S3). Comparisons among video laryngoscope types showed that C-MAC had higher success than TruView (RR 1.18, CI 1.02–1.37), while C-MAC versus MacGrath showed no significant difference (RR 1.03, CI 0.95–1.12; Figure S4). Both comparisons were rated as very low certainty of evidence (Table 2). Digital intubation demonstrated higher success compared with direct laryngoscope (RR 1.81, CI 1.18–2.76; Figure S5), though evidence was highly limited (Table 2).

**Table 2:** A modified summary of findings table and certainty of evidence assessments for the outcome of first attempt success rate in infant and neonate intubation for all comparisons

Comparison	N of studies	N of participants	Effect estimate (CI)	GRADE
Video vs direct	13	2,029	RR 1.13 (1.06–1.20)	High
C-Mac vs TruView	1	80	RR 1.18 (1.02–1.37)	Very low^a^
C-Mac vs MacGrath	1	138	RR 1.03 (0.95–1.12)	Very low^a^
Digital vs direct	1	45	RR 1.81 (1.18–2.76)	Very low^b^
Remifentanil vs morphine-midazolam	1	71	RR 1.09 (0.82–1.47)	Very low^a^
Remifentanil vs fentanyl	1	30	RR 1.50 (0.71–3.16)	Very low^a^
Relaxant vs no-relaxant	2	85	RR 2.31 (1.27–4.19)	Low^c^
Propofol vs morphine, atropine, and suxamethonium	1	53	RR 1.40 (1.00–1.96)	Very low^a^
Midazolame vs ketamine	1	60	RR 1.54 (1.12–2.13)	Very low^a^
Premedication vs awake	1	20	RR 2.25 (1.02–4.94)	Very low^a^
HFNC vs no support	2	294	RR 1.17 (0.92–1.48)	Moderate^d^
NIPPV vs no support	1	150	RR 1.15 (0.87–1.53)	Very low^a^
Any respiratory support vs no support	3	444	RR 1.18 (1.02–1.38)	Moderate^d^
Stylet vs no stylet	2	502	RR 1.10 (0.97–1.24)	Low^e^
C-curved stylet vs hockey stick stylet	1	114	RR 1.07 (0.99–1.16)	Very low^a^

^a^Downgraded three times due to imprecision as only one study included

^b^Downgraded three times due to imprecision as only one study included and downgraded due to study limitations (high risk of bias)

^c^Downgraded twice due to study limitations (high risk of bias) and only two small studies included

^d^Downgraded once due to limited number of included studies

^e^Downgraded twice due to limited number of included studies and study limitations (some concerns in risk of bias)

The certainty of evidence for pre-medication strategies were generally very low (Table 2; Figure S6). Two studies evaluating neuromuscular blocking agents (NMBA) reported higher success rates compared with no NMBA (RR 2.31, CI 1.27–4.19). Propofol use compared with morphine, atropine, and suxamethonium improved success (RR 1.40, CI 1.00–1.96) as did midazolam compared with ketamine (RR 1.54, CI 1.12–2.13). Premedication overall was associated with significantly higher success compared with intubations without the use of pharmacological agents (RR 2.25, CI 1.02–4.94).

HFNC use during intubation showed a trend toward improved first-attempt success rate (RR 1.17, CI 0.92–1.48, moderate certainty evidence, Figure S7). A rather similar effect size was observed in one study comparing NIPPV with no support (RR 1.15, CI 0.87–1.53; very low certainty evidence, Figure S7). When combined as any non-invasive respiratory support versus no support, the effect was statistically significant (RR 1.18, CI 1.02–1.38; Figure S7) with moderate-certainty evidence (Table 2).

Stylet use had slightly higher success rate when compared not using a stylet(RR 1.10, CI 0.97–1.24; Figure S8), and the same was observed when comparing C-curved stylet to hockey stick stylet (RR 1.07, CI 0.99–1.16; Figure S8). Certainty of evidence was rated as low and very low, respectively (Table 2).

### Time to successful intubation

Time to successful intubation was assessed in 11 studies comparing video laryngoscopy with direct laryngoscopy. In pooled analysis, no statistically significant mean difference (MD) was noted (− 0.1 s; CI − 4.9 to 4.8) (Figure S9). Sensitivity analysis including only low-risk-of-bias studies showed similar result (MD 1.4 s, CI − 2.2 to 5.0; Figure S10). Funnel plot inspection revealed no evidence of publication bias (Figure S11). Among video laryngoscope types, C-MAC versus TruView showed a statistically significant mean difference of − 5.0 s (CI − 8.4 to − 1.7). C-MAC versus MacGrath showed insignificant mean difference of − 1.0 s (CI − 3.7 to 1.7) (Figure S12). Digital intubation compared with direct laryngoscope demonstrated a mean difference was − 5 s (CI − 7.3 to − 2.7) (Figure S13).

Four studies assessed premedication strategies. Comparing remifentanil to morphine-midazolam resulted in a mean difference of − 6 s (CI − 16.1 to 4.8) (Figure S14). When remifentanil was compared with fentanyl, the mean difference was 15 s (CI − 175.2 to 205.2). Midazolam versus ketamine showed a difference of 3 s (CI − 8.1 to 14.3). The use of NMBA compared with no NMBA showed substantial reduction in intubation time (MD − 87 s (CI − 121.9 to − 52.1).

Time to successful intubation was reported in all the studies comparing respiratory support strategies. HFNC versus no support showed a mean difference of − 5.0 s (CI − 29.8 to 19.9) (Figure S15), and NIPPV versus no support showed − 8.0 s (CI − 21.5 to 5.50). All studies comparing different strategies for using stylets included time to successful intubation. Studies comparing stylet strategies showed no significant time outcomes when stylet versus no stylet was compared (MD − 0.1 s (CI − 4.9 to 4.7) (Figure S16). Comparison between C-curved stylet versus hockey stick stylet showed mean difference of − 13.0 s (CI − 20.6 to 5.4) (Figure S16).

### Desaturation

Desaturation outcomes were reported in 19 studies: 8 studies compared video versus direct laryngoscopy, 5 assessed premedication strategies, 1 compared video laryngoscope types, 1 evaluated HFNC and 1 NIPPV, and 2 compared stylet strategies. Thirteen studies reported number of desaturation events, two reported duration, and eight reported the lowest saturation (Table S2).

Definitions of desaturations varied considerably across studies, contributing to heterogeneity. In most studies, differences between intervention and control groups were small, though notable exceptions occurred. For example, NIPPV versus standard care showed desaturation in 32.0% of cases versus 57.3%, and stylet use versus no stylet showed 6.7% versus 19% (Table S2).

The duration of the desaturation was measured and reported in only two studies, with substantial variability due to differing measurement criteria. HFNC versus standard care showed no significant difference, whereas NIPPV versus standard care showed mean durations of 10 versus 20 s (Table S2).

The mean lowest saturation was measured in eight studies. Two studies comparing premedication strategies showed marked differences: Remifentanil was associated with a lowest recorded oxygen saturation of 47% compared with 35% for fentanyl. Another study comparing propofol with a combination of morphine, atropine and suxamethonium reported lowest saturations of 80% versus 60%, respectively (Table S2).

## Discussion

In this systematic review and meta-analysis, the use of video laryngoscope was associated higher first-attempt intubation success rate compared with direct laryngoscopy. Similar improvements were observed with non-invasive respiratory support and certain premedication strategies. Overall, video laryngoscopy improved success without prolonging intubation time. Non-invasive respiratory support appeared to enhance first attempt success rate and physiological safety during intubation. Different premedication strategies and intubation techniques showed modest benefits, but these findings were based on heterogeneous studies with lower certainty of evidence.

Video laryngoscopy clearly improved the first-attempt success compared with direct laryngoscopy, consistent with current international guidelines [10]. The 2025 European Resuscitation Council Guidelines recommend using a video laryngoscope for intubation whenever available [47] and the American Academy of Paediatrics and American Heart Association neonatal resuscitation guidelines also endorse its use [48]. Despite strong evidence and guideline support, adoption has been slow due to concerns about cost, cleaning logistics, and fears that direct laryngoscopy skills might decline [49] These concerns persist even though studies show that video laryngoscopy is particularly beneficial in low-volume units and does not compromise skill retention [50]. Given that neonates and small infants represent a high-risk group for airway management, prioritizing video laryngoscope availability for these patients is essential.

Non-invasive respiratory support during endotracheal intubation, including HFNC and NIPPV, showed promising results in improving first-attempt success rate and reducing desaturation. Although evidence remains limited and no formal guidelines exist, these findings suggest that pre-intubation HFNC is a low-risk, easily adoptable, minimally invasive intervention. A previous large randomized study in older children did find lower rate of hypoxemic events during emergency intubations when HFNC was used, but did not report statistically significant benefit in the intubation success rate [51] . However, as HFNC and NIPPV equipment is relatively costly, future research should include cost-effectiveness analyses to guide implementation.

Premedication strategies demonstrated potential benefits, but evidence certainty was low due to heterogeneity in study design, dosing, and provider experience. Current guidelines emphasize that no patient should be intubated without pharmacological support except during resuscitation.

Our findings reinforce the role of neuromuscular blocking agents (NMBA) in improving intubation success and reducing complications [52]. Modern reversal agents have minimized previous concerns about NMBA use in neonates and infants, reducing the rationale for avoiding these drugs. Future studies should explore standardized protocols for NMBA use in both elective and emergency settings.

Another important consideration is the variation in sample sizes across intervention categories. The evidence supporting video laryngoscopy was derived from the largest body of randomized trials and included over two thousand patients in pooled analyses, resulting in high-certainty evidence. In contrast, several other interventions, including pharmacological strategies, respiratory support during intubation, and stylet configurations, were evaluated in substantially fewer and smaller trials. These limited sample sizes contributed to wide confidence intervals and lower certainty ratings in the GRADE assessment.

### Strengths

A major strength of this review is the inclusion of all randomized controlled trials published on neonatal and infant intubation success. Including both neonates and infants enhances generalizability but introduces heterogeneity. Another strength was the strict adherence to protocol without deviations.

### Limitations

The main limitation is heterogeneity in settings, indications, and provider experience. The combination of acute and elective intubations causes notably heterogeneity to results. Intubating a neonate or infant in a controlled operating room differs substantially from emergency intubation in a delivery room, NICU or PICU. Provider experience ranged from novice pediatric residents to expert pediatric anesthesiologists, which likely influenced outcomes. Furthermore, the pooling of different noninvasive respiratory support modalities for the analyses most likely causes heterogeneity. Due to limited study numbers in most comparisons, we could not adjust for potential modifying factors except in video laryngoscopy analyses, where subgroup data have already been previously largely published [6]. A further limitation was the lack of grey literature search, which may predispose our results to possible publication bias. Another limitation was that the data was not completely dual extracted.

## Conclusion

High-certainty evidence supports video laryngoscopy as superior to direct laryngoscopy for first-attempt success. Moderate certainty evidence suggests non-invasive respiratory support during the endotracheal intubation also improves success. Time to successful intubation was similar across interventions. These findings strongly advocate prioritizing video laryngoscopy for neonatal and infant endotracheal intubations.

## Supplementary Information

Below is the link to the electronic supplementary material.

ESM 1 (31.8 KB DOCX)

## Data Availability

All data available in supplementary materials, and if something additional needed, available by contacting the corresponding author.
